# Robustness Fine-Tuning Deep Learning Model for Cancers Diagnosis Based on Histopathology Image Analysis

**DOI:** 10.3390/diagnostics13040699

**Published:** 2023-02-12

**Authors:** Sameh Abd El-Ghany, Mohammad Azad, Mohammed Elmogy

**Affiliations:** 1 Department of Information Systems, College of Computer and Information Sciences, Jouf University, Sakaka Al-Jouf 72341, Saudi Arabia; 2 Department of Computer Science, College of Computer and Information Sciences, Jouf University, Sakaka Al-Jouf 72341, Saudi Arabia; 3 Information Technology Department, Faculty of Computers and Information, Mansoura University, Mansoura 35516, Egypt

**Keywords:** histopathology images, colon cancer, lung cancer, ResNet101, fine-tuning

## Abstract

Histopathology is the most accurate way to diagnose cancer and identify prognostic and therapeutic targets. The likelihood of survival is significantly increased by early cancer detection. With deep networks’ enormous success, significant attempts have been made to analyze cancer disorders, particularly colon and lung cancers. In order to do this, this paper examines how well deep networks can diagnose various cancers using histopathology image processing. This work intends to increase the performance of deep learning architecture in processing histopathology images by constructing a novel fine-tuning deep network for colon and lung cancers. Such adjustments are performed using regularization, batch normalization, and hyperparameters optimization. The suggested fine-tuned model was evaluated using the LC2500 dataset. Our proposed model’s average precision, recall, F1-score, specificity, and accuracy were 99.84%, 99.85%, 99.84%, 99.96%, and 99.94%, respectively. The experimental findings reveal that the suggested fine-tuned learning model based on the pre-trained ResNet101 network achieves higher results against recent state-of-the-art approaches and other current powerful CNN models.

## 1. Introduction

The term “cancer” refers to a group of disorders in which the human body develops aberrant cells as a result of chance mutations. These cells multiply out of control as soon as they are produced, spreading throughout the organs. Any region of the body can produce cancerous cells, although the lungs, breasts, brain, colon, rectum, liver, stomach, skin, and prostate are the most frequently affected organs. Most cancers can eventually cause death if they are not treated. Cancer is the second leading cause of death worldwide, behind cardiovascular disorders. Worldwide, 9.958 million deaths from cancer and more than 19 million new cases were reported in 2020 [1]. The American cancer society forecasts that in 2022, more than 1.9 million new cancer cases will be recorded and more than 609,360 cancer-related deaths in the USA alone [2].

A variety of factors cause cancer, including physical carcinogens such as exposure to radiation and ultraviolet rays, behavioral features such as high body mass index and alcohol and tobacco use, as well as specific biological and genetic carcinogens [3]. The reason, nevertheless, may differ from patient to patient. Discomfort, exhaustion, nausea, persistent cough, breathing problems, weight loss, muscle pain, bleeding, bruises, and numerous other symptoms are typical cancer signs [4]. However, none of these signs are specific to cancer, nor do all patients experience them. Due to this, it might be challenging to identify cancer without a specific diagnostic technique such as computed tomography (CT), magnetic resonance imaging (MRI), positron emission tomography (PET), ultrasound, or biopsy.

Deep learning (DL)-based automated systems for diagnosing medical diseases, particularly cancer, have become commonplace in recent years. While many works that attempt to automate this diagnosis do so using CT and MRI scans [5,6,7], there are others. Deep learning techniques have considerably improved the early detection of breast cancer, for example, by utilizing dynamic contrast-enhanced magnetic resonance imaging (DCE-MRI). Similarly, deep learning has been applied to CT scans to identify lung cancer and classify images of brain tumors [8].

Lung and colon cancer diagnosis using DL has been a more popular study topic in recent years. Automated diagnosis has been employed in the majority of successful investigations with images from histopathology slides. This study only uses histopathology pictures to automatically detect lung and colon tumors. This work aims to categorize the photos of colon and lung cancer into five groups: squamous cell carcinomas, adenocarcinomas lung, benign lung, benign colon, and adenocarcinomas colon. It also aims to significantly enhance the DL-based prognosis of such malignancies by delivering superior outcomes. We have carried out substantial research using several DL models. The findings demonstrate that the model can classify related lung and colon cancer subtypes. The main contributions of this paper are as follows:

1. The ResNet101 model is fine-tuned to diagnose multi-type cancer lesions with high performance.
2. Transfer learning is used to train a benchmark cancer lesions dataset containing more than 25,000 histopathology images.
3. Five different metrics are used to evaluate the performance of the proposed model. Moreover, they are used to compare the performance of the proposed model with other state-of-the-art models and systems. The experimental results show that the proposed approach achieved promising results for diagnosing different cancer types.

The rest of the article is organized as follows. The most recent related research in this area is covered in Section 2. The proposed strategy for the early identification of lung and colon cancer is described in Section 3 and is based on many DL models. The experimental findings where the proposed approach is contrasted with the current state-of-the-art approaches are shown in Section 4. Section 5 ends the proposed research and offers suggestions for further improvement.

## 2. Related Work

The nature of medical imaging data has always affected researchers when developing diagnosis and prognosis systems based on deep learning [9]. The most common types of medical imaging data include MRIs, X-rays, CT scans, endoscopic images, and histopathological slides [10,11,12]. Despite the difficulty of the cancer detection and classification challenge, authors have employed deep learning techniques to significantly advance cancer detection systems [13].

Deep learning models can identify the most common malignancies, including breast cancer. For instance, researchers [14,15] have developed deep learning techniques to reasonably diagnose breast cancer. Similarly, authors [16,17] have employed DL techniques to identify bladder cancer. Another common form of cancer is skin cancer, which has seen few significant advances. For example, Jinnai et al. [18] proposed a DL algorithm to identify skin cancer. DL techniques have also been applied to the identification of cancer stem cell morphology [19], gastric cancer [20], and oral squamous cell carcinoma [21]. Although DL methods for categorizing and identifying lung and colon cancer employing histopathology images have gained popularity in recent years [22], little progress has been made to date [23] due to a lack of data. Amidst the paucity of data, a select few researchers have made substantial contributions [24]. While some authors primarily concentrate on colon cancer categorization [25], others focus exclusively on lung cancer classification [26]. Recent studies have attempted to simultaneously classify images of colon and lung cancer. The authors used prior-trained systems in a transfer learning environment or created and trained their originally developed systems from the start [27,28,29].

Few notable papers only classify lung tumors. For example, Abbas et al. [30] only classified lung cancers using prior-trained systems AlexNet, VGG-19, ResNet-101, ResNet-50, ResNet-34, and ResNet-18. They divided the images into three categories: benign-lung, squamous cell carcinoma-lung, and adenocarcinoma-lung. According to their claims, all prior-trained systems achieved an F1-score of 97.3%, 99.7%, 98.6%, 99.2%, 99.9%, and 99.9%, respectively. On the other hand, Roy et al. [31] used a capsule network to categorize photos of lung cancer histology. They said that a relatively simple setup allows them to achieve an average accuracy of 99%. Colon cancer has undergone a few significant categorization changes. Bukhari et al. [32] categorized colonic tissue using histological pictures using three convolutional neural networks (CNN): ResNet-18, ResNet-30, and ResNet50. They asserted that ResNet-50 achieved average accuracy equal to 93.91%, while ResNet-18 and ResNet-30 each obtained an accuracy of 93.04%.

A lung cancer diagnosis technique based on CNN plus the feature learning technique of nodule region of interest (ROI) was introduced by Suresh and Mohan [33]. They gathered CT scan pictures from the infectious disease research institute (IDRI) databases and the lung image database consortium (LIDC). They used generative adversarial networks (GANs) to create more images to expand the sample. They could attain a maximum classification accuracy of 93.9% using CNN-based classification methods. A lung nodule detection technique applied on CT scan images and utilizing a light CNN structure was described by Masud et al. [34]. When tested on the LIDC dataset, their model successfully distinguished benign, malignant, and normal cases with a classification accuracy of 97.9%. Another CT scan image-based lung cancer screening technique was put forth by Shakeel et al. They used an improved deep neural network (IDNN) for picture segmentation and several ensemble methods (EM) for image classification after eliminating noise from the images [35].

Masud et al. [27] developed a histological lung and colon picture classification method based on DL. They applied domain transformations of two types to extract four sets of characteristics for image classification. They then mixed the characteristics of the two categories to reach their classification conclusions. They claimed to have a 96.33% accuracy rate. Similarly, Mangal et al. [36] used a shallow neural network design to categorize histopathological images into five categories. They asserted that their research classified lung and colon cancers with 97% and 96% accuracy, respectively. Table 1 summarizes existing methods for colon and lung cancer prediction.

## 3. Materials and Methods

### 3.1. Dataset

**Figure 1:**
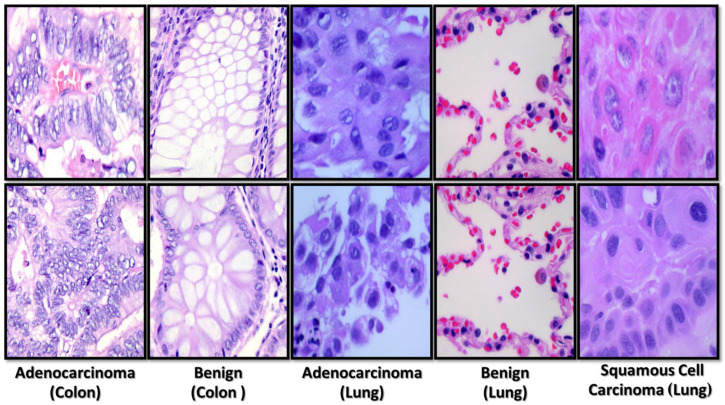
Some examples of histopathological images from the used dataset.

This paper used the histopathological images (LC25000) dataset created by A. Borkowski and his associates and published in 2020 [37]. This collection contains 25,000 photos of lung and colon tissues divided into five groups. It has 25,000 photos, of which 15,000 are of lung cancer and 10,000 are of colon cancer. Squamous cell carcinoma, benign, and adenocarcinoma are the three different forms of lung tissue pictures. In contrast, colon pictures fall under benign tissues and cancer groups. The LC25000 dataset was developed utilizing a sample of HIPAA-compliant. It verified references, including 750 lung tissue (250 adenocarcinomas, 250 squamous cell carcinomas, and 250 benign tissue) and 500 colon tissue (250 adenocarcinomas and 250 benign tissue) augmented to create 25,000 images. The dataset was increased by flipping and rotating the photographs under various conditions; as a result, the dataset was separated into five categories with 5000 images each. There are now 25,000 images in the dataset. Images were scaled to 224 × 224. Figure 1 shows samples of histopathological images from the dataset.

### 3.2. Model Architecture and Training

#### 3.2.1. Prior Processing

The preprocessing stage is applied to scale up and normalize the data before feeding the images to the model. The pixel density of the processed images ranges from high to low. Higher image values may produce different loss values from the lower range values. Therefore, it is necessary to normalize the dataset. The deep learning architecture scales the image pixels before the training stage. To harmonize image samples, image pixel values are normalized from [0, 255] to [0, 1]. Without scaling, a significant number of votes will be needed to decide how to update weights for the high-pixel range images [38].

#### 3.2.2. Training Procedure

A deep learning network can handle complex problems and improve classification/recognition accuracy [39]. However, there may be challenges when training the deep network, such as saturation, accuracy degradation, and disappearing or bursting gradients [40]. These issues can be resolved by utilizing deep residual pre-trained architecture. Pre-trained model architecture makes it easier to train deeper networks than the earlier deeper framework [39]. Resnet101 was previously trained using ImageNet, which has a total of 1.5 million photos of natural scenes [40]. ResNet has the ability to restructure network layers using leftover learning functions. The stacked layers in ResNet are a perfect fit for the intended mapping (residual mapping) [40].

**Figure 2:**
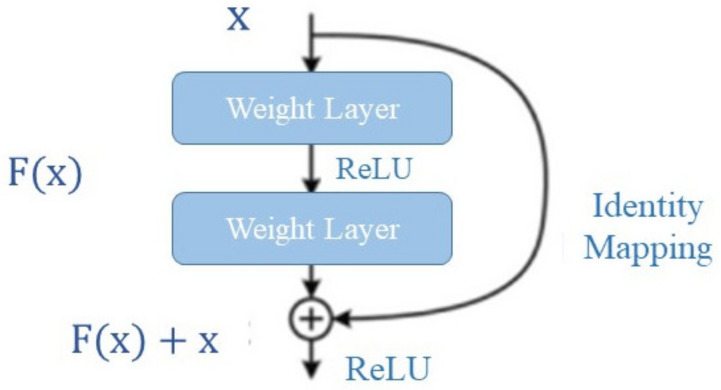
Illustration of ResNet101’s identity mapping.

ResNet101’s central premise is the identity mapping, as illustrated in Figure 2. It is used to forecast the essentials to arrive at the final prediction of the outputs from the preceding layer [40]. ResNet101 reduces the vanishing gradient phenomenon by taking a different shortcut. The model can pass through the extra layers due to identity mapping. This makes it easier for the model to avoid overfitting [41].

**Figure 3:**
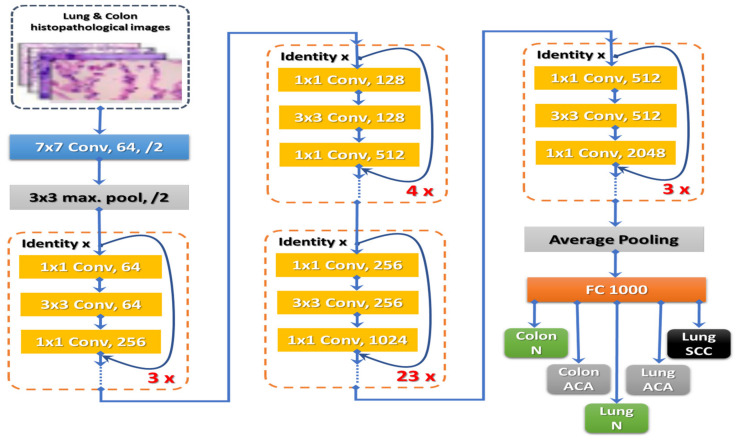
ResNet101’s architectural layout.

During training, the weights of the prior-trained ResNet101 model were used. Such a prolonged procedure makes it easier to train deeper networks and improves accuracy. Figure 3 depicts ResNet101’s architectural layout.

#### 3.2.3. Optimization of the Network

Since data have increased exponentially, optimization has become increasingly important, particularly in deep learning. The deep layer network’s extensive set of parameters makes it challenging to manage the difficulties in changing the network settings [42]. These optimization algorithms work to improve the outcomes by applying a variety of optimization strategies [43].

The model’s performance is affected by setting the hyper-parameter. Numerous effective strategies for automatically adjusting the hyper-parameters have been developed through optimization [44]. The learning performance rate is significantly influenced by the Adam optimizer’s optimization techniques [45]. It is necessary to tune the architecture to increase the performance, which is called fine-tuning [46,47]. This can be achieved by selecting a suitable network of deep learning. Furthermore, the choices of layers, hyper-parameters, and optimizers should also be included to achieve such tuning [40]. Transfer learning, batch normalization, hyper-parameter tuning, regularization, optimization via the Adam optimizer, and cross-entropy are fine-tuning techniques used in the proposed deep model.

##### Batch Normalization and Hyper-Parameter Tuning

For convolutional networks, batch normalization improves optimization performance [48]. Understanding the fixed input distributions could minimize the number of epochs needed, eliminate the impacts of the internal covariate shift, and reduce generalization error [49]. Batch normalization can be used to perform standardization by computing the average and standard deviation for each mini-batch of input data for a layer during training [50]. The average and standard deviation of activation are calculated to normalize features using Equations (1) and (2) [49].

(1) $${\bar{x}}_{f}=\frac{1}{m}\sum_{i=1}^{m}x_{if}$$

(2) $$\sigma_{f}=\frac{1}{m}\sum_{i=1}^{m}\left(x_{if}-{\bar{x}}_{f}\right)$$

where $x_{if}$ is the fth feature of the ith sample, and m is the size of a mini-batch. Equation (3) [49] allows for the normalization of features using the average and standard deviation of the mini-batch.

(3) $${\hat{x}}_{f}=\frac{x_{f}-{\bar{x}}_{f}}{\sigma_{k}+\xi }$$

where the modest positive constant ξ is used to provide numerical stability. Batch normalization employs two learnable parameters in practice as $\beta_{f}$ and $\gamma_{f}\;$for each feature f during the training phase [49].

(4) $$BN\left(x_{f}\right)=\gamma_{f}{\hat{x}}_{f}+\beta_{f}$$

The backpropagation approach updates training and adapts parameters in accordance with the transformed inputs. Batch normalization aims to increase the network’s stability by properly distributing the activation values during the training. Initializing weights before deep network training is a difficult problem. When training deep networks, the choice of weight initialization can be handled by achieving stability using batch normalization [50]. Batch normalization is a technique for data preparation that can be used to standardize raw input data with different scales [48].

##### Activation Function

Regression problems are examples of complicated transformations that cannot be learned through linear activation. As nonlinear activation functions such as sigmoid and hyperbolic tangents do not have linear behavior, nodes can learn more complex data structures [51]. The saturation of sigmoid and hyperbolic tangent functions is a prevalent issue. When z is close to 0, they are sensitive to input value and saturate to very high or very low levels for positive and negative values, respectively [52].

Using the sigmoid and tanh functions fails to provide adequate gradient information for deep layers in big networks. With more layers, the error utilized in backpropagation, which updates the weights over the network, becomes smaller [53]. As a result, deep networks are unable to properly learn or determine the proper direction of parameters to enhance the cost function [51]. This leads to the vanishing gradient problem.

Deep networks with deep layers must be trained using a specific activation function. In order to understand the complex relationships within the data and avoid simple saturation, this activation function must behave as a nonlinear function. However, it must also behave as a linear function in order to be responsive to the activation input total. To solve such problems, Rectified Linear Units (ReLU) were introduced. They replace hidden sigmoid units with piecewise linear hidden units [53].

a. ReLU

To all hidden layers, we applied a ReLU activation function. Three fully linked layers came after the max pooling layers. The dropout layer and softmax classifier are coupled at the final layers to achieve excellent training accuracy. Following the dropout, the results are connected and smudge-free due to the softmax. Convolutions, the ReLU, and batch normalization are all included in the feature mapping. The model is broken into numerous blocks with stacked layers to shrink the feature map’s size while keeping it constrained. As a result, the model eventually prepared the dataset for epochs of 14.

ReLU is a commonly used activation function in networks containing many layers. Numerous issues are solved by ReLU, including the vanishing gradient issue [53]. Below is the equation for ReLU:

(5) $$ReLU\left(Z\right)=\max\left(0,z\right)$$

We can update a specific weight using the following update rule:

(6) $$w^{*}=w+\eta \;\frac{{\delta E}^{2}}{\delta w}$$

where $\frac{{\delta E}^{2}}{\delta w}$ is the partial derivative of the error relative to w, η is the learning rate, and $w^{*}$ is the updated weight [54].

How sensitive the error $E^{2}$ is to the weight is explained by the error term’s derivation. The chain rule can be used to evaluate the derivative term. A vanishing gradient issue arises if relatively tiny modifications are made to the partial derivative results. The weight values increase quickly in the explosion task, contrary to the vanishing task [52]. If input z is less than zero, the ReLU activation function is set to zero; if it is equal to or larger than zero, it is set to z. ReLU helps build convolutional networks since it helps the model learn more rapidly [51].

b. Softmax

Softmax enables the system to associate particular classes with particular logits by enhancing logit values for the target classes. Additionally, it may offer a discrete probabilistic model of the class results [48]. This may result in a successful training procedure and the creation of a valuable machine learning model. In addition to its normalizing capabilities, softmax can be quite beneficial for optimizing the network model [54].

Vectors are compressed into the range of (0, 1) using the softmax function for all results or some. These vectors are seen as scores representing class likelihood in multiclass prediction [48]. The output scores should be written as s. Not only the $S_{i}$ classes separately, but also the complete class is necessary for the softmax function to work. The corresponding equation is described below:

(7) $$f\left(S_{i}\right)=\frac{e^{S_{i}}}{\sum_{j}^{c}e^{S_{j}}}$$

where $S_{j}$ is the score derived from the net for all classes. When no activation function is used, softmax ensures that the final network layer outputs have non-negative real-valued probabilities and an overall summation of one [48]. The forecasts and targets are compared via iterative procedures, and the results are compiled into a loss value. The gain for backpropagation is estimated using the loss value [55]. After that, the performance is improved by utilizing the optimizer and its quirks. The iterative processes end when the model significantly improves its performance [48].

##### Optimization

The goal of optimization tasks is to search the optimum mapping function $f\left(x\right)$ that minimizes the loss function L of the training rows of number N [42],

(8) $$\;\underset{\theta }{\min}\frac{1}{N}\sum_{i=1}^{N}L\;\left(y^{i},f\left(x^{i},\theta \right)\right)$$

where $x^{i}$ is the feature vector for the ith sample, $y^{i}$ is the matching label, and θ is the parameter of the mapping function.

Stochastic gradient descent (SGD) performs better for large-scale data than batch gradient descent [43]. SGD eliminates calculation redundancy and reduces updating times for big data samples. Instead of computing the gradient’s value during iterations, SGD updates the gradient using just one random sample. The SGD has the ability to converge more quickly, and its cost is independent of the sample size [42]. This is how the loss function in the equation might be expressed [45]:

(9) $$L\left(\theta \right)=\frac{1}{N}\sum_{i=1}^{N}\frac{1}{2}{\left(y^{i},f_{\theta }\left(x^{i}\right)\right)}^{2}=\frac{1}{N}\sum_{i=1}^{N}cost\;\left(\theta ,\left(x^{i},y^{i}\right)\right)$$

For a randomly chosen sample i in SGD, the loss function L is as follows [42]:

(10) $$L^{*}\left(\theta \right)=\frac{1}{2}{\left(y^{i},f_{\theta }\left(x^{i}\right)\right)}^{2}=cost\;\left(\theta ,\left(x^{i},y^{i}\right)\right)$$

In SGD, the gradient is updated using a random sample i rather than all samples in each iteration [45]:

(11) $$\overset{´}{\;\theta }=\theta +\eta \left(y^{i},f_{\theta }\left(x^{i}\right)\right)x^{i}$$

where $\overset{´}{\theta }$ is the update of the gradient depending on the preceding update, and η is the learning rate. The Adaptive Gradient Method (AdaGrad) is a simple improvement to SGD. AdaGrad dynamically modifies the learning rate utilizing previous iterations. The following is the gradient update for AdaGrad [45]:

(12) $$\theta_{t+1}=\theta_{t}+\eta \frac{g_{t}}{V_{t}}$$

where $V_{t}$ is the total historical gradient of parameter θ at step t, $g_{t}$ is the gradient of parameter θ at step t, η is the learning rate, and $\theta_{t}$ is the value of parameter θ at step t. AdaGrad enhancement is used to compute the second-order cumulative momentum to address the radically decreasing learning rates [45].

(13) $$\;V_{t}=\sqrt{\beta \;V_{t-1}+\left(1-\beta \right){\left(g_{t}\right)}^{2}}\;$$

where β denotes the exponential decay parameter. The SGD technique adds a new advancement with adaptive moment estimation (Adam). Adam integrates the adaptive learning approach with the momentum methods and provides an adjustable learning rate for each parameter [44]. As with the momentum approach [45], Adam stores the average of the exponential decay of the past squared gradients $m_{t}$, rather than the average of the exponential decay of the past squared gradients $V_{t}$.

(14) $$m_{t}=\beta_{1}\;m_{t-1}+\left(1-\beta_{1}\right)g_{t}$$

(15) $$V_{t}=\sqrt{\beta_{2}\;V_{t-1}+\left(1-\beta_{2}\right){\left(g_{t}\right)}^{2}}\;$$

where the exponential decay rates are $\beta_{1}$ and $\beta_{2}$. As a result, Equation (16) [33] provides the parameter θ’s ultimate form.

(16) $$\theta_{t+1}=m_{t}-\eta \;\frac{\sqrt{1-\beta_{2}}}{1-\beta_{1}}\;\frac{m_{t}}{V_{t}+\epsilon }$$

Most implementations use 0.9, 0.999, and $10^{-8}$ as the default values for $\beta_{1}$, $\beta_{2}$, and ϵ, respectively. Compared to similar adaptive learning rate algorithms, Adam performs better in practice [42]. An extension of Adam is adaptive max pooling (AdaMax), which can be used to reduce network errors and improve performance.

Another important aspect of optimization is the selection of the loss function. The current model state can be regularly estimated using the model’s loss function [56]. The weights might be updated in a suitable manner to lessen the loss on the subsequent evaluation depending on the chosen loss function [57].

The cross-entropy loss function is frequently utilized when solving multiclass classification problems with provided integer target values. The goal integer values assigned in experiments are regarded as categorical [42]. The cross-entropy score is calculated based on the average difference between actual and anticipated values across all classes. This score is decreased up to the optimal cross-entropy score of 0. In Equation (17), categorical cross-entropy L is defined [57].

(17) $$L=\prod_{c=1}^{C}y_{c}{\left(x,w_{c}\right)}^{t_{c}}$$

where $y_{c}$ is the output-based input x and weight $w_{c}$, c is the index running over the classes number, and $t_{c}$ is the number of occurrences of c. Mathematically, this function is assessed using the maximum likelihood approach to inference. Maximizing the likelihood of the training set is achieved by minimizing the loss, as in Equation (18) [57].

(18) $$L=-\sum_{c=1}^{C}y_{c}\;.\log{\hat{y}}_{c}$$

where log indicates log-likelihood, ${\hat{y}}_{c}$ is the corresponding model output, and $y_{c}$ is the corresponding target value. Using this function for a prediction problem rather than the sum of squares yields better generalization and training results [56].

## 4. Model Implementation and Evaluation

### 4.1. Hardware and Software Specifications

Kaggle is used in experiments to speed up GPU-focused deep learning applications. Nvidia K80 GPU, 12 GB RAM, and 2496 CUDA cores comprise the hardware setup for Kaggle’s accelerated runtime, which is used to run the written program.

### 4.2. Model Implementation

The LC25000 dataset’s histopathology images were initially scaled down to 224 × 224 and sampled for scale augmentation. Then, in order to create new data, affine picture modifications, including rotation, shifting, scaling (zoom in/out), and flipping, were combined. Before each activation and after each convolution, batch normalization was used. Additionally, different batch sizes were used (40 and 80). The model underwent 15 training iterations. Epsilon was set at 0.001, momentum to 0.99, and weight decay to 0.0001. The initial learning rate was set to 0.001, and the error rate reached a plateau. The learning rate dropped by 0.5. Ninety percent of the dataset’s images were used for training, and 10% were used for testing and validation. Different hyper-parameter methods were used to achieve the best results, including regularization and optimization utilizing the AdaMax and SGD optimizers and the categorical cross-entropy loss function. All used hyper-parameters that achieved the highest performance for the tested model are listed in Table 2.

### 4.3. Performance Evaluation

The metrics used to evaluate the performance of the proposed fine-tuning model are shown in Equations (19)–(23).

(19) $$\mathrm{Precision}=\frac{\mathrm{TP}}{\mathrm{TP}\;+\;\mathrm{FP}\;}$$

(20) $$\mathrm{Recall}=\frac{\mathrm{TP}}{\mathrm{TP}\;+\;\mathrm{FN}}$$

F-Score = (2 × Precision × Recall)/(Precision + Recall) (21)

(22) $$\mathrm{Spec}=\frac{\mathrm{TN}}{\mathrm{TN}\;+\;\mathrm{FP}\;}$$

(23) $$\mathrm{Accuracy}=\frac{\mathrm{TP}+\mathrm{TN}}{\left(\mathrm{TP}\;+\;\mathrm{FP}\;+\;\mathrm{TN}\;+\;\mathrm{FN}\;\right)}\times 100$$

where TP represents the number of correctly labeled positive occurrences, FP represents the number of incorrectly labeled positive instances, TN represents the number of correctly labeled negative instances, and FN represents the number of incorrectly labeled negative instances.

### 4.4. Experimental Results

The LC25000 dataset, which consists of 25,000 histopathology images of five distinct classes, was divided into 90% for training, 5% for validation, and 5% for testing for the first run. The model was trained by using 14 epochs. The AdaMax optimizer was used for the first training run, while the SGD optimizer was used for the second trial. Six performance indicators were computed separately for each class in the proposed framework to assess its performance. As a result, these values’ averages were calculated.

#### 4.4.1. Analysis of ResNet101 Model

The analysis of our proposed model was carried out with different activation functions, optimizers, and batch sizes. Table 3 shows the suggested model’s performance using different hyper-parameters, including optimizers, activation functions, and batch sizes. Six performance metrics were used to record the performance: precision, recall, F-score, specificity, accuracy, and Cohen Kappa. Additionally, the test time was recorded to compare the speed of each hybrid of the parameters. As illustrated in Table 3, the proposed model achieved the highest performance measures: precision, recall, F-score, and specificity, when using the AdaMax optimizer. The ReLU activation function and batch size is 80, achieving precision equals 99.70%, recall equals 99.68%, F-score equals 99.68%, and specificity equals 99.91%. The best accuracy is (99.89%). This was achieved when using the SGD optimizer and ReLU activation function. The best Cohen Kappa is 100%, which was achieved using batch size 40, optimizer AdaMax, and activation function ReLU.

**Figure 4:**
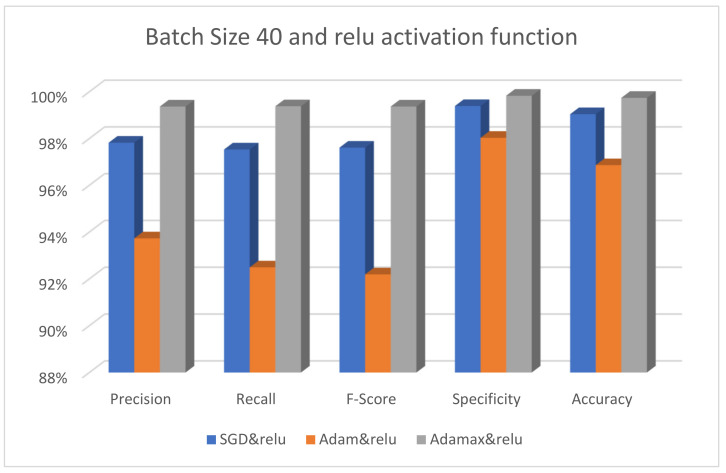
The performance of the ResNet101 model with different optimizers using batch size 40 and the ReLU activation function.

**Figure 5:**
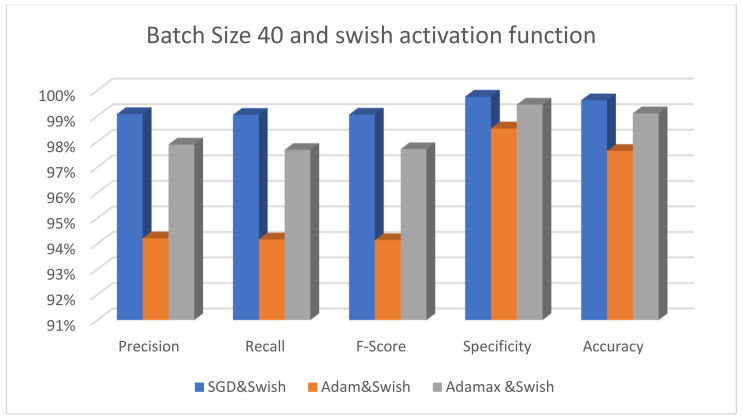
The performance of the ResNet101 model with different optimizers using batch size 40 and the Swish activation function.

Figure 3 displays the performance of the suggested model using three different optimizers—SGD, Adam, and AdaMax—while keeping Swish as the activation function and 40 as the batch size. Figure 4 displays the effectiveness of the suggested model using the same varied optimizers with the activation function changed only to ReLU. Figure 5 shows that the SGD optimizer provides the best performance compared to other optimizers. Adam has the lowest accuracy results since it requires many hyperparameters and iterations. Moreover, it is sensitive to feature scaling.

**Figure 6:**
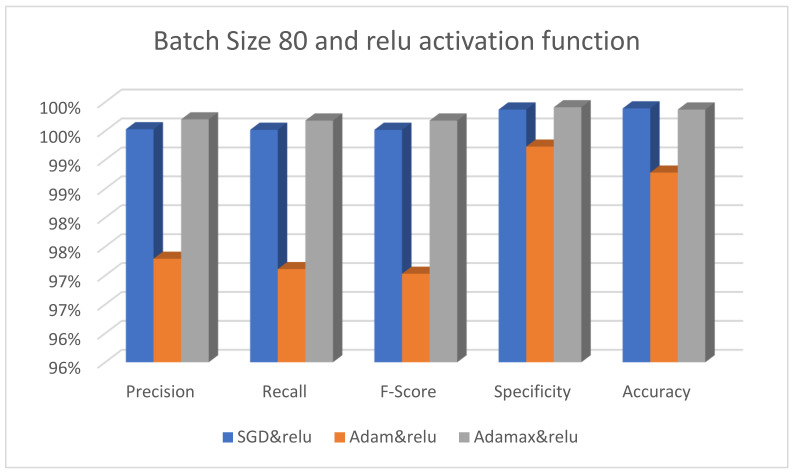
The performance of the ResNet101 model with different optimizers using batch size 80 and the Swish activation function.

The effectiveness of the suggested model using different optimizers is shown in Figure 5. The SGD, Adam, and AdaMax optimizers have their batch size fixed at 40 and the activation function set to ReLU. It is obvious that the AdaMax optimizer performs better than the other optimizers. Adam also has the worst results when the ReLU activation function is used. Figure 6 displays the performance of the suggested model with the same optimizers, the batch size set to 80, and the activation function fixed at Swish. It is clear that the SGD optimizer performs well, whereas the Adam optimizer yields the poorest results.

**Figure 7:**
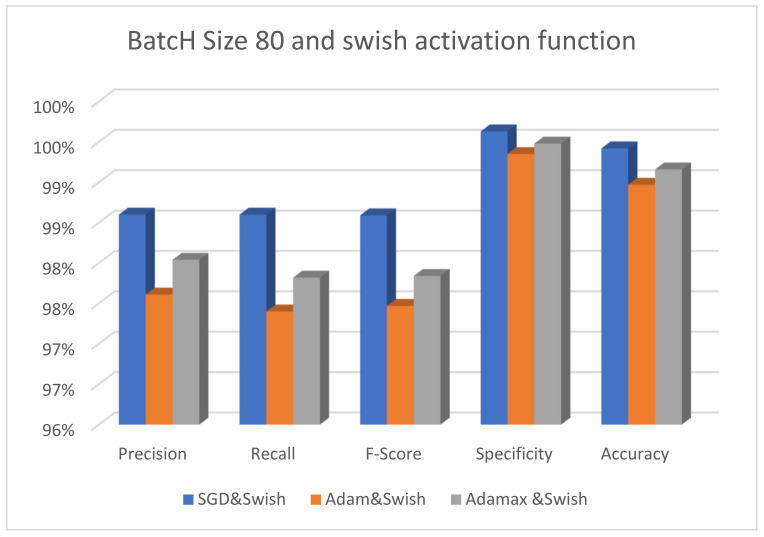
The performance of the ResNet101 model with different optimizers using batch size 80 and the ReLU activation function.

The effectiveness of the suggested model using various optimizers is shown in Figure 7. The SGD, Adam, and AdaMax optimizers have their batch size fixed at 80 and the activation function set to ReLU. It is obvious that the AdaMax optimizer performs better than the other optimizers. Adam also notes the worst performance when the batch size is 80 and the ReLU activation function is used.

From Figure 4, Figure 5, Figure 6 and Figure 7, it is concluded that the optimizer SGD will work with the activation function Swish, while the AdaMax optimizer will work with the ReLU activation function. In contrast, the Adam optimizer is not affected by the activation function change.

**Figure 8:**
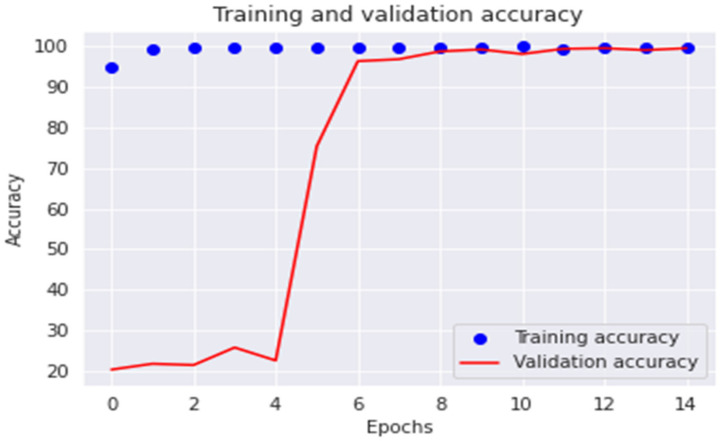
The ResNet101 model accuracy vs. epoch numbers.

**Figure 9:**
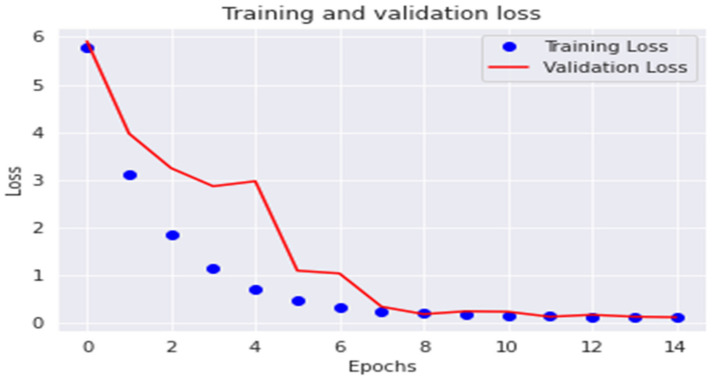
The ResNet101 model loss vs. epoch numbers.

The accuracy and loss are displayed in Figure 8 and Figure 9 for every epoch of the training and validation sample. As shown in Figure 8, the model accuracy is constant and roughly comparable to the training and validation datasets after epoch 4. Furthermore, between epochs 7 and 14, the model loss is significantly reduced and almost equal for both the training and validation sample, as shown in Figure 9. This suggests that the proposed model is free of the well-known overfitting issue.

**Figure 10:**
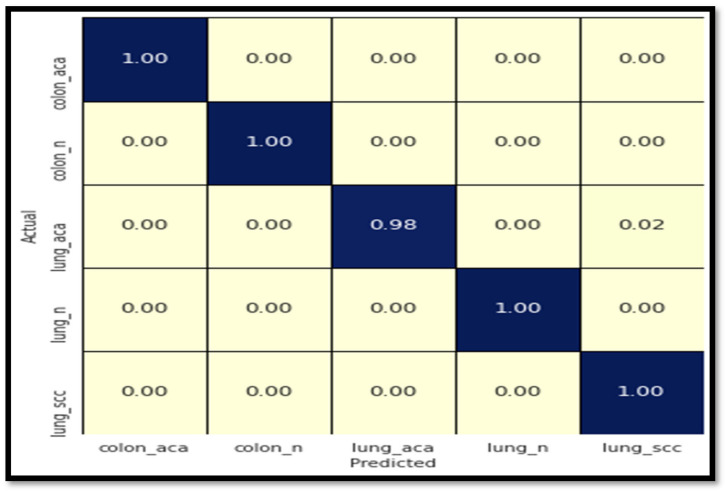
The confusion matrix for colon and lung cancer class classification using the ResNet101 model.

Figure 10 shows the confusion matrix for colon and lung cancer classes using the ResNet101 model classification problem. Figure 10 clarifies that all photos of colon cancer are correctly classified, whereas photographs of lung cancer are incorrectly classified. Just 2% of lung_aca photos were incorrectly categorized as lung_scc images. It is, therefore, fantastic that additional lung cancer photos were incorrectly categorized under different disease groups. Apparently, the network confuses lung cancer images with colon cancer images.

#### 4.4.2. Comparison with Other Four Powerful Deep Learning Models

Table 4 compares the proposed model’s average classification performance to four well-known DL models for the classification problem. It shows that MobileNet, Xception, InceptionV3, and Resnet101 have average accuracy levels greater than 99%. Resnet101model has the highest average accuracy (99.94%). On the other hand, VGG16 achieves the lowest average accuracy (97.63%). The highest F-score (99.84%), highest recall (99.85%), best precision (99.84%), and highest specificity (99.96%) are all produced by the Resnet101 model. It is also noticed that the VGG16 model attains the lowest performance metric, achieving the lowest precision (94.22%), recall (94.08%), F-score (94.06%), and specificity (98.52%).

#### 4.4.3. Comparison of the Proposed Model and the State-of-Art Methods

Table 5 shows a comparative analysis of the proposed model and state-of-the-art methods. As indicated in the last row in Table 5, our proposed fine-tuned ResNet model outperforms other state-of-the-art methods in all performance metrics.

#### 4.4.4. Discussion

Deep learning approaches employ pre-trained convolutional neural network models to detect lung and colon cancer. A fine-tuning model for lung and cancer detection is presented in this paper. The outcome demonstrates that the suggested model significantly enhances multi-type cancer detection performance, particularly for histopathology slide images.

Early research predictions of colon and lung cancer frequently occurred independently. They categorize images of lung and colon cancer using pre-trained models. Lung and colon cancer are treated independently as a binary classification problem. Although all binary classification tasks yielded respectable results, this does not necessarily mean that these models are ready for use in practical situations. Our method differs from earlier systems in that it uses a fine-tuning model. Our method simultaneously classifies lung and colon cancer photos using a multiclass technique.

Our findings suggest that the suggested model could be applied to lung and cancer detection histopathological image analysis. Table 4 lists the classification accuracy and methods used in earlier studies that used the same LC25000 dataset. Deep learning-based methodologies have typically produced successful results in past studies. However, the proposed model is as accurate as older state-of-the-art techniques. The classification of cancerous tissues using many raw data sets produced by merging various datasets would have more clinical value and produce more accurate results. The limitations and shortcomings of the proposed paradigm will be the main topics of our future work.

## 5. Conclusions

Due to the huge success of deep networks, major efforts have been made to research cancer problems, particularly colon and lung cancer. The potential of deep learning in the multi-classification of seven main colon and lung lesions was studied in this work. We examined a range of fine-tuning strategies for the improvement of diagnostic performance. The pre-trained ResNet101 network outperforms other deep learning models in performance evaluation using LC25000 histopathology images (25,000 total). The model is fine-tuned using regularization, batch normalization, and hyperparameter optimization. The Adam optimizer and cross-entropy loss function are also used with ideal settings. By contrasting MobileNet, Xception, VGG16, and InceptionV3, four strong models—including the suggested fine-tuned deep model—were assessed. The suggested model demonstrates that fine-tuning models perform better than current strong techniques in terms of diagnostic accuracy. Future work will test the proposed model on different types of human cancers and use hyper-optimization algorithms to obtain better hyper-parameterization automatically.

## Figures and Tables

**Table 1:** Summary of existing methods for colon and lung cancer prediction.

Study	Methodology	Obtained Results	Dataset	Limitation
Sakr et al. [25]	CNN with four convolution block	Accuracy = 99.5%	LC25000	Only colon cancer
Masud et al. [27]	Multi-channel CNN	Accuracy = 96.33%	LC25000	Custom architecture
Mangal et al. [36]	Multi-channel CNN	Accuracy = 97.89%	LC25000	
Abbas et al. [30]	VGG-19, Alex Net, ResNet: ResNet-18, ResNet-34, ResNet-50, and ResNet-101	F-1 scores = 0.973, 0.997, 0.986, 0.992, 0.999, and 0.999, respectively	LC25000	Only lung cancer
Roy Medhi [31]	Capsule network	Accuracy = 99%	LC25000	Only lung cancer
Bukhari et al. [32]	ResNet-18, ResNet-30, and ResNet50	ResNet-50 accuracy = 93.91% ResNet-30 accuracy = 93.04% ResNet-18 accuracy = 93.04%	LC25000	Only colon cancer

**Table 2:** Values of the hyper-parameters used in the Resnet101 model architectures.

Hyper-Parameters	Value	The Best Value
Number of epochs	14	14
Batch size	40/80	80
Activation function	Swish/ReLU	ReLU
Optimizer	Adam/AdaMax/SGD	AdaMax
Initial learning rate	0.001	0.001
Dropout	0.5	0.5
Patience	10	10
Loss function	Categorical cross-entropy	Categorical cross-entropy

**Table 3:** The performance of the proposed model using different hyper-parameters.

Batch	Parameters	Precision (%)	Recall (%)	F-Score (%)	Specificity (%)	Accuracy (%)	Cohen Kappa (%)	Test_Time/Step
Batch size 40	SGD&Swish	99.07	99.04	99.05	99.75	99.61	98.39	5 s 215 ms
	Adam&Swish	94.21	94.16	94.14	98.51	97.63	91.99	7 s 213 ms
	Adamax&Swish	97.88	97.67	97.70	99.44	99.10	96.40	7 s 213 ms
	SGD&ReLU	97.82	97.53	97.61	99.39	99.04	96.00	8 s 239 ms
	Adam&ReLU	93.73	92.49	92.19	98.03	96.86	89.24	8 s 244 ms
	Adamax&ReLU	99.37	99.38	99.37	99.83	99.74	**100.00**	7 s 227 ms
Batch size 80	SGD&Swish	98.60	98.60	98.59	99.63	99.42	97.60	5 s 198 ms
	Adam&Swish	97.61	97.40	97.47	99.35	98.97	97.20	7 s 232 ms
	Adamax&Swish	98.04	97.82	97.84	99.48	99.16	97.20	7 s 219 ms
	SGD&ReLU	99.53	99.52	99.52	99.87	**99.89**	99.60	7 s 224 ms
	Adam&ReLU	97.29	97.11	97.03	99.23	98.78	98.00	7 s 217 ms
	**Adamax&ReLU**	**99.70**	**99.68**	**99.68**	**99.91**	99.87	99.60	6 s 232 ms

**Table 4:** Average classification results for the classification task.

	Precision (%)	Recall (%)	F1-Score (%)	Specificity (%)	Accuracy (%)
MobileNet	99.52	99.52	99.52	99.88	99.81
Xception	99.61	99.20	99.60	99.60	99.84
VGG16	94.22	94.08	94.06	98.52	97.63
InceptionV3	99.60	99.60	99.60	99.90	99.84
Resnet101	99.84	99.85	99.84	99.96	99.94

**Table 5:** The comparison of the proposed model and the state-of-the-art methods.

Reference	Methodology	Performance
Bukhari et al. [30]	ResNet-18, ResNet-30, and ResNet-50	ResNet-50 accuracy = 93.91% ResNet-30 accuracy = 93.04% ResNet-18 accuracy = 93.04%
Roy Medhi [29]	Capsule network	Accuracy = 99%
Abbas et al. [28]	VGG-19, Alex Net, ResNet: ResNet-18, ResNet-34, ResNet-50, and ResNet-101	F-1 scores = 0.973, 0.997, 0.986, 0.992, 0.999, and 0.999, respectively
Sakr et al. [36]	CNN with four convolution block	Accuracy = 99.5%
Masud et al. [25]	Multi-channel CNN	Accuracy = 96.33%
Mangal et al. [34]	Multi-channel CNN	Accuracy = 97.89%
The proposed model	Fine-tuned ResNet101	Precision (99.84%), recall (99.85%), F-Score (99.84%), specificity (99.96%), and accuracy (99.94)

## Data Availability

The dataset is open access and available at the following link: https://www.kaggle.com/datasets/andrewmvd/lung-and-colon-cancer-histopathological-images (Last access on 11 February 2023).
